# Funikuläre Myelose und Polyneuropathie durch Lachgasinhalation – eine Differenzialdiagnose des Guillain-Barré-Syndroms

**DOI:** 10.1007/s00115-023-01443-1

**Published:** 2023-02-17

**Authors:** Julius N. Meißner, Katharina Hill, Asadeh Lakghomi, Louisa Nitsch

**Affiliations:** 1 Klinik und Poliklinik für Neurologie, Universitätsklinikum Bonn, Venusberg Campus 1, 53127 Bonn, Deutschland; 2 Klinik für Neuroradiologie, Universitätsklinikum Bonn, Bonn, Deutschland

## Hintergrund

Der inhalative Konsum von Lachgas ist mit einer Lebenszeitprävalenz von etwa 11 % in Deutschland weit verbreitet [2]. Der Gebrauch führt dosisabhängig zu einem metabolischen Vitamin‑B_12_-Mangel, welcher sich klinisch in einer Polyneuropathie und einer funikulären Myelose äußern kann [7]. Wir berichten hier über einen Fall von lachgasinduzierter Polyneuropathie und funikulärer Myelose, welcher klinisch einem Guillain-Barré-Syndrom ähnelte.

## Fallbeispiel

**Figure 1:**
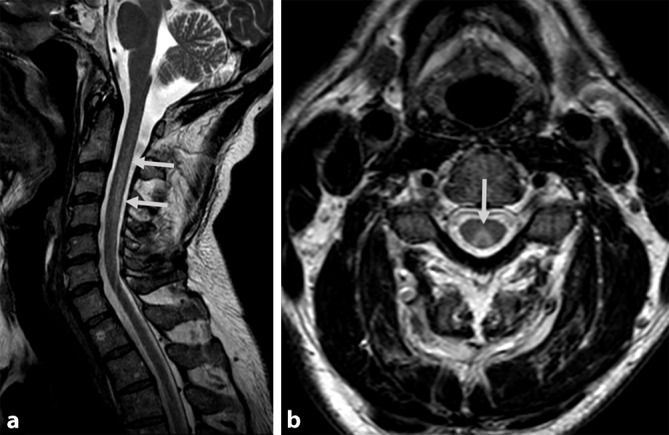

Ein 45-jähriger Mann wurde uns mit vier Wochen zuvor erstmals aufgetretenen, progredienten Akroparästhesien und symmetrisch aufsteigenden Paresen vorgestellt. Er berichtete auf Nachfrage über die Inhalation von vier mit Lachgas gefüllten Luftballons sieben Wochen zuvor. Es bestand eine ausgewogene Ernährung, eine spezielle Diät wurde nicht eingehalten. In der neurologischen Untersuchung fielen eine sensible Ataxie mit distal symmetrischer Hypästhesie und eine Pallhypästhesie bei erhaltenem Temperaturempfinden auf. Die Achillessehenenreflexe waren ausgefallen und es zeigten sich Paresen der distalen Extremitätenmuskeln. Es fand sich keine autonome Dysfunktion und keine Hirnnervenausfälle. Die Liquordiagnostik erbrachte unauffällige Befunde mit normwertigem Eiweißgehalt, normwertiger Zellzahl sowie unauffälliger Lactat- und Glukosekonzentration, sodass kein Anhalt für eine Infektion bestand. Eine Borrelienserologie war unauffällig. Gangliosidantikörper (GM1, GQ1b, und GD1b, jeweils IgG und IgM) waren im Serum nicht nachweisbar. Labordiagnostisch fiel eine leicht reduzierte Erythrozytenzahl von 4,2 T/l (Normbereich 4,3–5,75 T/l) bei leichter hyperchromer (MCH 34 pg; Normbereich 27–33,5 pg) Makrozytose (MCV 102 fl; Normbereich 80–99 fl) bei normwertiger Hämoglobinkonzentration auf. Die Vitamin‑B_12_- und Holotranscobalamin-Serumspiegel lagen im Normbereich. Allerdings fiel eine schwere Hyperhomocysteinämie von 108,12 µmol/l (Normbereich 3,2–10,7 µmol/l) auf. Die Elektroneurographie erbrachte den Befund einer axonal-demyelinisierenden Polyneuropathie mit verlängerten F‑Wellen-Latenzen, verlängerten distal motorischen Latenzen, herabgesetzten Nervenleitgeschwindigkeiten und reduzierten Muskelsummenaktionspotenzialen (Tab. 1). Eine zervikale spinale Magnetresonanztomographie zeigte T2-Hyperintensitäten der Hinterstränge in den Höhen C2 bis C7, welche in der sagittalen Bildgebung V‑förmig zur Darstellung kamen (Abb. 1a, b).

**Table 1:** 

*Nerv* Stimulationsort	Latenz (ms)	F‑Wellen-Latenz (ms)	Amplitude (mV)	Nervenleitgeschwindigkeit (m/s)
**Motorisch**				
*N. medianus rechts*				
Handgelenk	3,44 (≤ 4,5)	31,4 (≤ 32)	7,3 s (≥ 3)	**–**
Ellenbeuge	9,43	–	6,4 (≥ 3)	**43 **(≥ 47)
*N. peroneus rechts*				
Knöchel	**5,42** (≤ 5)	–	3,3 (≥ 3)	–
Fibulakopf	14,9	**–**	**2,8** (≥ 3)	**34 **(≥ 40)
Kniekehle	16,7	**–**	**2,7** (≥ 3)	49 (≥ 40)
*N. tibialis links*				
Knöchel	5,47 (≤ 5,8)	**64,0** (≤ 60)	1,1 (≥ 3)	**–**
Kniekehle	18,13	–	0,9 (≥ 3)	**37 **(≥ 40)
*N. tibialis rechts*				
Knöchel	–	**64,5** (≤ 60)	–	**–**
**Sensibel**				
*N. medianus rechts*				
Zeigefinger	–	–	**1,9** (≥ 3)	**42** (≥ 43)
*N. ulnaris rechts*				
Kleinfinger	–	–	3,2 (≥ 3)	41 (≥ 41)
*N. suralis rechts*				
Knöchel	–	–	**0,5** (≥ 3)	**38** (≥ 43)

In der motorischen Neurographie des N. medianus rechts herabgesetzte mNLG (motorische Nervenleitgeschwindigkeit). N. peroneus rechts mit herabgesetzter mNLG bei Stimulation unterhalb des Fibulakopfes, reduzierten MSAP (motorische Summenaktionspotenziale) ober- und unterhalb des Fibulakopfes und verlängerter DML (distal motorische Latenz). N. tibialis links mit herabgesetzter mNLG, reduzierten MSAP und verlängerte F‑Wellen-Latenz. N. tibialis rechts mit verlängerter F‑Wellen-Latenz. In der sensiblen Neurographie des N. medianus rechts und N. suralis rechts herabgesetzte sNLG (sensible Nervenleitgeschwindigkeit) und reduzierte SNAP (sensible Nervenaktionspotenziale). Regelrechte sensible Neurographie des N. ulnaris rechts

Die neurographischen Auffälligkeiten sprechen für eine sensomotorische axonal-demyelinisierende Polyneuropathie

Auf Basis der erhobenen Befunde wurde die Diagnose einer durch Lachgasinhalation induzierten funikulären Myelose und Polyneuropathie gestellt. Eine hochdosierte Substitution mit Vitamin‑B_12_ wurde eingeleitet. Bereits in den ersten Tagen nach Therapiebeginn kam es zu einer Besserung der Beschwerden.

## Diskussion

Die Inhalation von Lachgas führt zu einem metabolischen Vitamin‑B_12_-Mangel, welcher neben Blutbildveränderungen zu einem neurologischen Syndrom mit Polyneuropathie und Hinterstrangaffektion führen kann [7]. Die symmetrischen Veränderungen der Hinterstränge werden als „inverted V-sign“ beschrieben [3]. Neurographisch können sowohl Zeichen eines axonalen Schadens als auch einer Demyelinisierung nachweisbar sein. Die routinemäßig bestimmten Vitamin‑B_12_- und Holotranscobalamin-Serumspiegel sind weniger sensitiv als die Bestimmung des Homocysteinspiegels und der Methylmalonsäure, weshalb der metabolische Vitamin‑B_12_-Mangel leicht übersehen werden kann [4]. Ein normaler Vitamin‑B_12_-Spiegel wird bei 20–40 % der Patienten beschrieben. Auf die Bestimmung von Methylmalonsäure wurde verzichtet, weil die Bestimmung von Holotranscobalamin und Methylmalonsäure in etwa den gleichen Stellenwert bei Patienten mit Lachgasinhalation einnehmen (Methylmalonsäure 93,8 % vs. Homocystein 90,3 % auffällige Werte; [4]). Ein vorbestehender Vitamn‑B_12_-Mangel stellt dabei ein Risikofaktor für die Entwicklung neurologischer Symptome nach Lachgasinhalation dar [6]. Der zugrunde liegende Pathomechanismus ist eine durch Lachgas induzierte Oxidation des Cobalt-Zentralions des biologisch aktiven Methylcobalamins und Inaktivierung von Adenosylcobalamin, welche zu einer Inhibition der Enzyme Methylmalonyl-CoA-Mutase und Methioninsynthase führt, deren Substrate (Methylmalonsäure bzw. Homocystein) dann akkumulieren. Die Neurotoxizität ist unter anderem Folge eines hierdurch verursachten Methioninmangels, der eine Myelinisierungsstörung verursacht [1]. Zu beachten ist, dass die klinische Präsentation und die neurographischen Veränderungen einem Guillain-Barré-Syndrom ähneln können [5]. Der vorgestellte Fall unterstreicht daher die Bedeutung einer sorgfältigen Suchtmittelanamnese bei Patienten mit subakut aufgetretener Polyneuropathie.

## Fazit für die Praxis

- Die Inhalation von Lachgas kann zu einem metabolischen Vitamin‑B_12_-Mangel führen, welcher sich in einer Polyneuropathie und funikulären Myelose äußern kann.
- Neben Vitamin‑B_12_ und Holotranscobalamin sollten auch weitere Parameter wie der Spiegel von Homocystein oder Methylmalonsäure bestimmt werden.
- Eine konsequente Substitution von Vitamin‑B_12_ und der Verzicht auf weitere Lachgasinhalation sind entscheidend für die Therapie.
- Eine Suchtmittelanamnese sollte bei allen Patienten mit subakuter Polyneuropathie erfolgen.
